# The Physician’s Bedside Record with Dietary

**Published:** 1894-06

**Authors:** 


THE PHYSICIAN'S BEDSIDE RECORD, WITH DIETARY. By Gideon C. Segur, M. D. The Plympton Manufacturing Co., Hartford, Conn. Price, 10 cents each, or $1.00 per dozen.

This little pamphlet contains blank sheets ruled for record of patients in long illnesses. There are columns for time of observations, pulse, temperature, respiration, medicine given, notes of the nurse, and at bottom space for written directions of the attending physician. There are temperature and pulse charts, and a printed dietary for the more simple food preparations. Each book is intended for one patient, and each page for one day's history of the patient's case. The book is small—about the size of an ordinary pass book, and is exceedingly convenient. It would be very useful in hospitals.

